# An overview of psoriatic arthritis – epidemiology, clinical features, pathophysiology and novel treatment targets

**DOI:** 10.1007/s00508-016-1111-9

**Published:** 2016-11-07

**Authors:** Andreas Kerschbaumer, Karl H. Fenzl, Ludwig Erlacher, Daniel Aletaha

**Affiliations:** 1 Department of Internal Medicine III, Division of Rheumatology, Medical University of Vienna, Währinger Gürtel 18-20, 1090 Vienna, Austria; 2 Karl Landsteiner Institute for Autoimmune Diseases and Rheumatology, Kundratstraße 3, 1100 Vienna, Austria; 3 2nd Medical Department with Rheumatology, Osteology and Geriatrics, Kundratstraße 3, 1100 Vienna, Austria

**Keywords:** Psoriatic arthritis, Clinical aspects, Epidemiology, Treatment, Pathophysiology

## Abstract

Psoriatic arthritis is a chronic inflammatory joint disease occurring in a subgroup of patients suffering from psoriasis. This article gives an overview of the complexity of psoriatic arthritis, looking at several aspects of this heterogeneous disease, such as epidemiology, important clinical features and comorbidities as well as current concepts of the pathophysiology and subsequent insights in novel treatment targets.

## Epidemiology

A meta-analysis carried out by Alamanos et al. found a wide variation in the annual incidence of psoriatic arthritis (PsA) ranging from 0.1 to 23.1 cases per 100,000 (median 6.4/100,000) inhabitants with large differences between countries. The mean age at diagnosis varied between 40.7 and 52.0 years (median 47.7 years). Prevalence rates also varied between 1 case per 100,000 (Japan) to 420 per 100,000 (Italian) inhabitants [1]. Depending on the definitions used (e.g. diagnostic codes, patient self-reporting, rheumatologist diagnosis and classification criteria) prevalence and incidence rates vary substantially. Prevalence rates of PsA in psoriasis (Pso) patients range from 6 % to 41 % [2].

## Clinical features

The presence of Pso, inflammatory arthritis and absence of positive serological tests for rheumatoid arthritis (RA) are the hallmarks of PsA. In 60–70 % of patients Pso precedes PsA, while in 15–20 % arthritis precedes the onset of Pso. In a small group of patients (15–20 %) the two manifestations appear within 1 year. Asymmetrical oligoarthritis is the most common joint pattern at disease onset [3]. Regarding joint involvement, five groups of inflammatory joint patterns were defined by Moll and Wright: (1) distal interphalangeal (DIP) predominant arthritis, (2) arthritis mutilans, (3) symmetrical polyarthritis, (4) asymmetrical oligoarthritis or monoarthritis and (5) ankylosing spondylitis predominance [4]. Moll and Wright proposed asymmetrical oligoarthritis as the most frequent clinical joint pattern of PsA. Studies regarding the joint pattern distribution in PsA patients vary, partly because of different definitions of PsA by different researchers and partly due to the fact that there is a probable change of the joint pattern with increasing disease duration [5]. Axial PsA, with typical features, such as asymmetrical sacroiliitis, nonmarginal and asymmetrical syndesmophytes, paravertebral ossification and involvement of the cervical spine can be manifested in a broad variety of symptoms [6]. Dactylitis is the clinical term for diffuse inflammation and swelling of a whole finger or toe and represents a cardinal finding in PsA patients [4]. The pathophysiological correlate is the combination of flexor tenosynovitis, joint effusion and subcutaneous edema, which is difficult to distinguish clinically and the diagnosis can therefore be supported by magnetic resonance imaging (MRI) or ultrasound examinations [7]. Inflammation at tendon, ligament, joint capsule sites and fascia insertion sites into bone is called enthesitis and is another hallmark feature of PsA. Pain and swelling in affected areas are very common and can be found in approximately half of PsA patients. Typical entheseal inflammation sites are the Achilles tendon, the plantar fascia, the greater trochanter tubercle of the femur, the medial femur condyles and epicondyles of the olecranon [8]. Last, but not least, nail involvement is much more common with PsA patients than with Pso patients without arthritis [4]. Clinical presentation of nail changes includes nail pitting, transverse ridging, yellowish discoloration in onycholysis, subungual hyperkeratosis, splinter hemorrhages and even total destruction of the nail [4, 9].

Of special importance are extra-articular manifestations (EAM) of PsA. They are much more common than previously thought and seem to be associated with axial disease. The first study exclusively focusing on EAM in PsA by Peluso et al. in 2015 could demonstrate an EAM prevalence of 49 % in a retrospective analysis of 387 PsA patients. Most were male patients with axial disease and a significantly longer disease duration than PsA patients without EAM [10]. The most common EAM affect the eyes, the gastrointestinal tract, the heart and arteries and the urogenital system and are summarized in Table 1.

**Table 1:** Extra-articular manifestations of PsA

Gastrointestinal	Ocular	Cardiovascular	Urogenital
Crohn’s disease	Uveitis	Bundle branch blocks	Urethritis
Ulcerative colitis	Conjunctivitis	Intraventricular blocks	Prostatitis
Non-specific colitis		Increased arterial stiffness	Balanitis
		Increased carotid media thickness	Cervicitis
			Vaginitis

## Comorbidities

The most important comorbidities associated with PsA are those of cardiovascular diseases. An increased prevalence of cardiovascular risk factors in PsA patients was shown in several studies and PsA seems to be associated with obesity, hypertension, insulin resistance, type II diabetes and hyperlipidemia. Vascular comorbidities include ischemic heart disease, hypertension, dyslipidemia, atherosclerosis, peripheral vascular disease and cerebrovascular disease. The pathophysiological link seems to be a shared inflammatory pathway of both metabolic syndrome and PsA [11, 12]. A large cohort study in the United Kingdom found an increased incidence of diabetes in PsA, which may be explained by obesity and lifestyle factors [13]. Metabolic syndrome and insulin resistance are highly prevalent in PsA patients and there is a strong association to the severity of inflammatory disease [14, 15]. Thyroid autoimmunity is increased in PsA patients, reflected in increased antibodies against thyroid peroxidase and a hypoechoic thyroid on ultrasound examination, especially in female patients [16].

## Pathophysiology

The occurrence of PsA is almost certainly immune-mediated and probably shares pathogenic mechanisms with Pso. The PsA synovium shows infiltration with T cells, B cells and macrophages. Clonally expanded CD8+ T cells are frequent in PsA. Plasmacytoid dendritic cells are thought to play a key role in Pso and there is some evidence that they are also involved in PsA. The extensive bone lesions in PsA are consistent with the findings of osteoclastic progenitors in peripheral blood of PsA patients, as well as upregulation of receptor activator of nuclear factor kappa b ligand (RANKL) in the synovial lining layer. Cytokines derived from Th17 are likely to be important in PsA, given their prominence in Pso and in other forms of spondylarthritis [17]. Innate immunity also seems to play a role in the pathogenesis of PsA [18].

### Concepts of pathogenesis

Two major hypotheses regarding the pathogenesis of PsA are under discussion and in the scientific focus at the moment. One hypothesis considers PsA as a classical autoimmune disease, the other as a disease originating from inflammation primarily occurring in the entheseal organ after trauma or physical stress [19–21].

#### Classical autoimmune disease

Looking at a classical autoimmune mechanism, with autoreactive CD8+ T cell clones inducing inflammation after binding a self-peptide through major histocompatibility complex (MHC) class I molecules, PsA shows certain genetic and immunological features to support this hypothesis. There is evidence for a susceptibility to develop PsA in association with certain MHC class I genes [22]. The observation of CD4+ T cell depletion and persistence of disease in human immunodeficiency virus (HIV) positive PsA patients suggest that PsA is driven by autoreactive CD8+ T cells, activated through presentation of a self-peptide by MHC class I susceptibility molecules [23]. Additionally, the absence of autoantibodies and predominance of CD8+ T cells in joint fluid and in synovial tissue supports the claim that PsA is an MHC class I-associated and CD8+ T cell-mediated autoimmune disease [20]. A relationship of genotype and determining phenotypes was proposed by FitzGerald et al. in 2015 [20]; therefore, of particular interest may be the possible pathogenetic explanation of Moll and Wright’s initial proposed subtypes of PsA with entheseal, synovial and axial predominant forms [4].

#### Enthesitis as primary site of inflammation

In 1998 McGonagle et al. proposed the hypothesis that synovitis in spondylarthropathies may be secondary to entheseal inflammation, occurring as an epiphenomenon of proinflammatory cytokines and growth factors from the enthesitis [21]. Subsequent anatomical studies could show that enthesis, initially declared to be the insertion site of a muscle or a tendon into bone, is not that simple to define. The entheseal structure and physiology seem to be more complex and the definition of an entheseal organ, including enthesis and surrounding tissues, such as bursae, periosteal fibrocartilage, synovial covered fat pads and sesamoid bones would fit better in terms of complexity [24, 25]. The close anatomical relationship between synovium and enthesis is especially prone to mechanical stress. Microtrauma-induced secretion of immune mediators seems to promote an altered vascularity of entheses in elderly, healthy individuals and may furthermore induce inflammation in predisposed patients, leading to clinically significant inflammation of joints and development of spondylarthropathy or PsA [26, 27]. This may also be part of the explanation of an epidemiological association of higher body mass index (BMI) with PsA development, with increased mechanical stress on entheseal structures due to greater body weight [25].

Further evidence supporting this hypothesis is the initial site of inflammation in spondylarthropathy model-related animal models. In 2014 Jacques et al. could demonstrate a mechanotransduction-associated origin of enthesitis and new bone formation at entheseal sites in TNF^ΔARE^ mice through activation of mitogen-activated protein kinase (MAPK) and Erk1/2 signalling pathways [28].

### Microbiome

As described in Table 1 an increased risk for developing Crohn’s disease is evident as a possible EAM of PsA. Furthermore, a higher incidence of subclinical gut inflammation has also been described in a subgroup of PsA patients [29]. A decreased bacterial diversity in PsA patients in comparison to Pso patients and healthy individuals has also been observed [30]. Gut dysbiosis might therefore also be a potential modulator of autoimmunity.

### Obesity

Obesity is an independent risk factor for development of PsA, as described in the section on risk factors. Low-grade chronic inflammation is associated with obesity and occurs in many organs and not limited to adipose tissue. White adipose tissue is an endocrine active organ secreting soluble mediators, which are responsible for a proinflammatory environment [31, 32]. Weight loss intervention in Pso patients receiving biologic agents resulted in an increased efficacy of biologic agents compared to controls [33]. In a recent review about autoimmune effects of obesity, Versini et al. concluded that the proinflammatory state through increased production of many adipokines including interleukin (IL)-6, tumor necrosis factor (TNF) alpha, resistin and leptin, aggravates the development of PsA and its comorbidities and decreases the efficacy of biologic therapy [32].

### Interleukin 23/Th17 pathway

In PsA patients, a new population of immune cells, IL-17+CD8+ T‑cells, were recently discovered in synovial fluid where IL-17+CD4-T-cell levels are increased in PsA synovial fluid in contrast to RA patients. These cells are comprised mainly of CD8+ cells, are positively correlated with disease activity (acute phase reactants, clinical and radiographic signs of active inflammatory joint disease) and may represent an important subpopulation of immune cells in the pathophysiology of PsA [34]. In Pso, IL-23 was found to be fundamentally important in pathogenesis and is mainly synthesized by dendritic cells of the dermis and macrophages, which is the main trigger for IL-17 production in the skin, initiating skin inflammation and acanthosis. Furthermore, pathogen products may directly stimulate the production of IL-17 [35]. Additionally, the treatment response of patients induced by inhibition of this pathway with biologic agents, such as ustekinumab (anti IL-12/23, European Medicines Agency EMA approval 01-2009), secukinumab (anti IL-17A, EMA approval 01-2015), ixekizumab (anti IL-17A, EMA approval 04-2016) and brodalumab (anti IL-17R, EMA approval pending) further underlines its importance in the pathogenesis of PsA [36].

## Novel treatment targets

In PsA therapy, conventional disease-modifying anti-rheumatic drugs (cDMARD) are commonly used in newly diagnosed diseases [37]. While evidence from large, well-designed clinical trials is missing, methotrexate, leflunomide and sulfasalazine are still regarded as first choice if treatment with non-steroidal anti-inflammatory drugs (NSAIDs) is insufficient. Current guidelines favor methotrexate as the first choice cDMARD, which could show better response than NSAIDs alone, especially regarding swollen and tender joints [38]. Leflunomide was shown to be significantly superior to placebo, with effects on peripheral arthritis and other PsA manifestations, such as pain, fatigue, dactylitis and skin disease [39]. Apremilast is a new, orally administered, selective inhibitor of phosphodiesterase 4 (PDE4). The inhibition of PDE4 increases cyclic adenosine monophosphate, downregulates the inflammatory cascade and leads to inhibition of Th1, Th2 and Th17 cytokines. Its clinical efficacy could be demonstrated in phase 3 trials by improving signs and symptoms of PsA as well as physical function [40, 41]. Ustekinumab is a fully human monoclonal immunoglobulin G1 antibody that binds the common p40 subunit of IL-12 and IL-23. Inhibition of IL-12 and IL-23 leads to an inhibition of Th1 and Th17 T‑cells leads to an inhibition of important inflammatory pathways in PsA [36]. In phase 3 trials ustekinumab especially showed improvement in skin disease but other factors, such as enthesitis, dactylitis, nail disease, physical function and quality of life also improve [42]. Another new therapeutic agent is secukinumab, a human monoclonal IgG1k antibody, targeting IL-17A, which also has a proinflammatory role in the pathophysiology of PsA. Recent phase 3 trials in PsA patients showed good efficacy of secukinumab in anti-TNF naive patients as well as patients who had previously received anti-TNF agents [43].

## Conclusion

Psoriatic arthritis resembles a complex inflammatory joint disease and this review tries to shed light on different aspects of PsA. Besides the psoriatic skin disease, which can also be difficult to diagnose in the first place or even be absent in the beginning of the course of PsA, the clinician has to focus on arthritis of peripheral joints, as well as axial disease, dactylitis and enthesitis. PsA is a very heterogeneous disease with a wide variety of extra-articular manifestations involving ocular, gastrointestinal, vascular, metabolic and urogenital symptoms. Comorbidities, such as the whole spectrum of the metabolic syndrome and thyroid autoimmunity, add additional complexity to PsA management. Our scientific insight into the pathophysiology of PsA is enlarging and implications on novel treatment options are evident. In this review, we hope to highlight some important aspects of PsA and simultaneously sensitize clinicians to the complexity of this heterogeneous disease.

### Conflict of interest

A. Kerschbaumer, K.H. Fenzl, L. Erlacher and D. Aletaha declare that they have no competing interests.
